# Tissue-friendly dentin treatments as a potential element in revascularization protocol (ex-vivo study)

**DOI:** 10.1186/s12903-025-05550-0

**Published:** 2025-02-03

**Authors:** Hala Fayek Khalil, Nadia Ezz El Din Metwalli, Sara Magdy, Mohamed Shamel

**Affiliations:** 1 Department of Endodontics, Faculty of Dentistry, The British University in Egypt, El Shorouk City, PO Box 43Suez Road, Cairo, 11837 Egypt; 2 Department of Pediatric Dentistry and Dental Public Health, Faculty of Dentistry, The British University in Egypt, El Shorouk City, Egypt; 3 Department of Orthodontics and Pediatric Dentistry, Faculty of Dentistry, The British University in Egypt, El Shorouk City, Egypt; 4 Department of Oral Biology, Faculty of Dentistry, The British University in Egypt, El Shorouk City, Egypt

**Keywords:** Dentine Treatments, Revascularization, Dual rinse HEDP, Curcumin, Cytotoxicity, Cell viability, SEM, Human Periodontal Stem Cells

## Abstract

**Background:**

Endodontic treatment aims to eliminate pulp tissue, microorganisms, and toxins while creating an environment conducive to tissue revitalization and regeneration. Sodium hypochlorite, the gold-standard irrigant, is effective but has significant cytotoxic effects, prompting the need for safer alternatives. This study investigates the cytotoxicity, cell proliferation, adhesion to dentin, and osteogenic differentiation of cells exposed to Dual Rinse HEDP, curcumin, and sodium hypochlorite (2.5%) for 1, 5, and 15 min, focusing on their potential application in revitalization and regenerative endodontic protocols.

**Methodology:**

Samples were assigned to groups based on the irrigant used: control, HEDP, curcumin, or sodium hypochlorite (2.5%) for exposure durations of 1, 5, and 15 min. Cytotoxicity was assessed using the MTT assay, with optical density measured at the specified times. Cell proliferation was evaluated via the Trypan blue exclusion test, with viable cells counted using a hemocytometer. Data were presented as mean and standard deviation (SD) values and statistical significance was set at *p* < 0.05 for all tests. Cell adherence to dentin discs was analyzed using scanning electron microscopy (SEM) after 5-min irrigant exposure. Osteogenic differentiation was assessed through alizarin red staining for calcium deposition and quantitative PCR analysis of BMP-2, TGF-β1, VEGF, and DSPP gene expression.

**Results:**

Cell cytotoxicity differed significantly across groups (*p* < 0.05), with HEDP showing the best results at 1 and 5 min. After 15 min, Group II had the highest value, followed by Group I. HEDP also recorded the highest cell proliferation, followed by curcumin. HEDP exhibited substantial calcium deposition and significantly upregulated BMP-2, TGF-β1, VEGF, and DSPP gene expression, surpassing other materials. Curcumin moderately promoted calcified nodule formation. Osteogenic media also induced significant gene upregulation.

**Conclusions:**

Dual Rinse HEDP and curcumin are tissue-friendly. Dual rinse HEDP efficiently increases stem cell adherence to dentin discs and their osteogenic differentiation.

So, this irrigant has the potential to be used in regeneration protocols.

## Background

Endodontic treatment goals include eliminating pulp tissue and microorganisms and reducing toxins from the radicular space. Root canal treatment includes shaping, cleaning, and obturation [1]. Banchs and Trope proposed the regenerative endodontic procedure in 2004. Revascularization and revitalization are used interchangeably in the literature. Both names stand for biological procedures to mimic the restoration of tooth tissues, such as the pulp, dentin, and root formation [2]. Regenerative medicine includes combining cells, enhancing materials, and essential growth factors. Regenerative endodontics became an alternative to treating nonvital teeth in which new pulp tissue is developed from undifferentiated cells [3].

Root canal disinfection of immature teeth for regenerative endodontics is not easy, as minimal endodontic instrumentation is needed to avoid root thickness loss causing the weakening of the tooth. Hence, incomplete bacterial eradication may happen. Agents employed in regenerative endodontics protocols (REPs) must have a broad-spectrum antibacterial activity to decontaminate the canals and promote stem cell attachment, proliferation, and differentiation. The protocol for regenerative endodontic procedures issued by the American Association of Endodontists advises the use of 1.5% sodium hypochlorite irrigant, then applying calcium hydroxide Ca(OH)_2_ as an intracanal medication or low concentrations antibiotic pastes (1–5 mg/ml), and 17% EDTA during the subsequent visit. However, these antimicrobial drugs may have adverse effects on stem cells [4].

Irrigation should follow mechanical preparation to ensure cleansing [1]. Several different components are used for root canal irrigation [5, 6]. An idyllic root canal irrigant should have no cytotoxic effect, acts broadly on microbes, can dissolute necrotic pulp tissue, bacterial toxins inactivator, and retard the establishment of a smear layer or help dissolve it [5, 7–10]. When extruded from the apical foramen, the irrigating solutions contact the periradicular tissues. If irrigation solutions are not biocompatible, they will cause the disintegration of cell proliferation, adhesion, and enzyme systems in the area of contact and cause periodontal tissue irritation, damage, degeneration, and delayed wound healing [11, 12]. Human periodontal ligament fibroblast cells (HPDLFc) are the first cells that face the emerging irrigation solutions from the root canal and are acknowledged as the primary cells responding to endodontic material in the periapical tissues [13, 14].

Dental materials can be tested biologically on 3 levels: primarily the toxicity checking of the dental material, then on animals to estimate the soft and hard tissue of the host, and lastly, simulate clinical practice. Pre-clinical biocompatibility assessment of materials used in dentistry is indispensable [15]. The cytotoxicity of an irrigation solution on HPDLFc is recognized from the median lethal concentration (LC50), which presents the material causing 50% cell culture death [16]. A material is toxic if it results in less than 50% of living cells after exposure [5, 17, 18]. One cytotoxicity test method is the enzymatic test using (3- (4.5-dimethylthiaziol-2yl) -2.5- diphenyl-tetrazolium bromide (MTT) assay. The MTT test is based on the mitochondrial activities of cells, so it assesses the ability of living cells. An irrigation solution is toxic if the percentage of living cells after being subjected to a material is lower than 50% [5, 15–18].

Sodium hypochlorite (NaOCl) (0.5%–6.15%) is the most used irrigation, acting broadly on microbes and liquefying organic tissues. However, the quest continues for a replacement due to its high toxicity [10, 19]. NaOCl is the preferred irrigant in most REP research because of its extensive antibacterial spectrum and tissue-dissolving capabilities. Numerous in vitro investigations have demonstrated that NaOCl exerts a concentration-dependent influence on the viability of SCAPs, with 6% NaOCl markedly diminishing stem cell survival. Consequently, a reduced concentration of NaOCl is advised in REPs. EDTA 17% demonstrated efficacy in mitigating the detrimental effects of NaOCl on stem cell adhesion, viability, differentiation, and the enhancement of endogenous growth factor release from dentin. Consequently, AAE and ESE recommendations advocate the application of 1.5% to 3% NaOCl, succeeded by 17% EDTA during the initial visit and 17% EDTA during the subsequent REPs visit [20].

The "soft" chelator HEDP (1-hydroxyethylidene-1,1-diphosphonic acid), also called etidronic acid, can be combined with NaOCl to provide a one-step chelating, disinfecting, and deproteinizing irrigant that eliminates the need for repeated rinses with incompatible chemicals. In terms of chelating power, HEDP is less forceful than EDTA. Continuous application of the one-step HEDP and NaOCl solution decreases the accumulation of hard tissue debris in the isthmus area by preventing the creation of a smear layer during instrumentation through a continuous chelation cycle.

The 0.9 gm etidronate powder in the Dual Rinse capsule (9% HEDP) (Medcem, GmbH, Weinfelden, Switzerland) must be combined with 10 mL of fresh NaOCl solution before use [21]. It was discovered that Dual Rinse HEDP added a little decalcifying element to the NaOCl solution. The duration required for adequate root canal irrigation is diminished when NaOCl and Dual Rinse HEDP are utilized concurrently as a single irrigating solution. The duration needed for adequate root canal irrigation is diminished when NaOCl and Dual Rinse HEDP are used concurrently as a singular irrigating solution. This facilitates chemical cleansing of the root canal and conditioning of the dentin for the later root canal fillings [22]. Clinically, Dual Rinse HEDP maintains NaOCl activity for an hour [23].

Curcumin, a chief ingredient in curries in India, China, Turkey, and Korea, is a natural polyphenol derived from Curcuma longa (turmeric). Curcumin is an antioxidant and antimicrobial agent. Curcumin also has curative abilities in diseases associated with inflammatory/immune responses like rheumatoid arthritis, pancreatitis, and diversity of cancers; it also urges osteoblastogenesis and hinders osteoclastogenesis in vitro and showed encouraging disinfection potentials, which may be helpful in endodontics [24].

Disinfection and the influence of irrigation solutions on cellular behavior are essential for a successful regenerative procedure [25, 26]. To discover an ideal biocompatible irrigant, the current study was designed to investigate sample cytotoxicity, cell proliferation, stem cell adhesion to treated dentin samples, and osteogenic differentiation of cells after contact with the studied irrigants Dual rinse HEDP, curcumin, and sodium hypochlorite 2.5% and for different observation periods of 1,5, 15 min.

This study hypothesizes that HEDP and curcumin, due to their biocompatibility and regenerative potential, will outperform NaOCl in cytotoxicity reduction, stem cell adhesion, and osteogenic differentiation. The novelty lies in exploring the combined effects of these agents, with potential applications in revitalization protocols to enhance treatment outcomes.

## Materials and methods

### Ethical committee approval

The cytotoxicity of the tested solutions was evaluated on cultured HPDLFc at the Faculty of Dentistry, Ain Shams University, Cairo, Egypt. Through grant CB project ID (21747) from the Science, Technology & Innovation Funding Authority (STDF), the study made use of the modern facilities of the Central Lab of Stem Cells and Biomaterial Applied Research (CLSBAR). Osteogenic differentiation and detecting osteogenic and odontogenic markers through RT-qPCR were performed at the Centre of Health Sciences, Dental Research Group, Faculty of Dentistry, British University in Egypt. Scanning electron microscopy (SEM) imaging was performed at the Nanotechnology Center, British University in Egypt. The British University in Egypt's Faculty of Dentistry's Research Ethics Committee approved the study protocol (FD BUE REC 21–032).

**Fig. 1:**
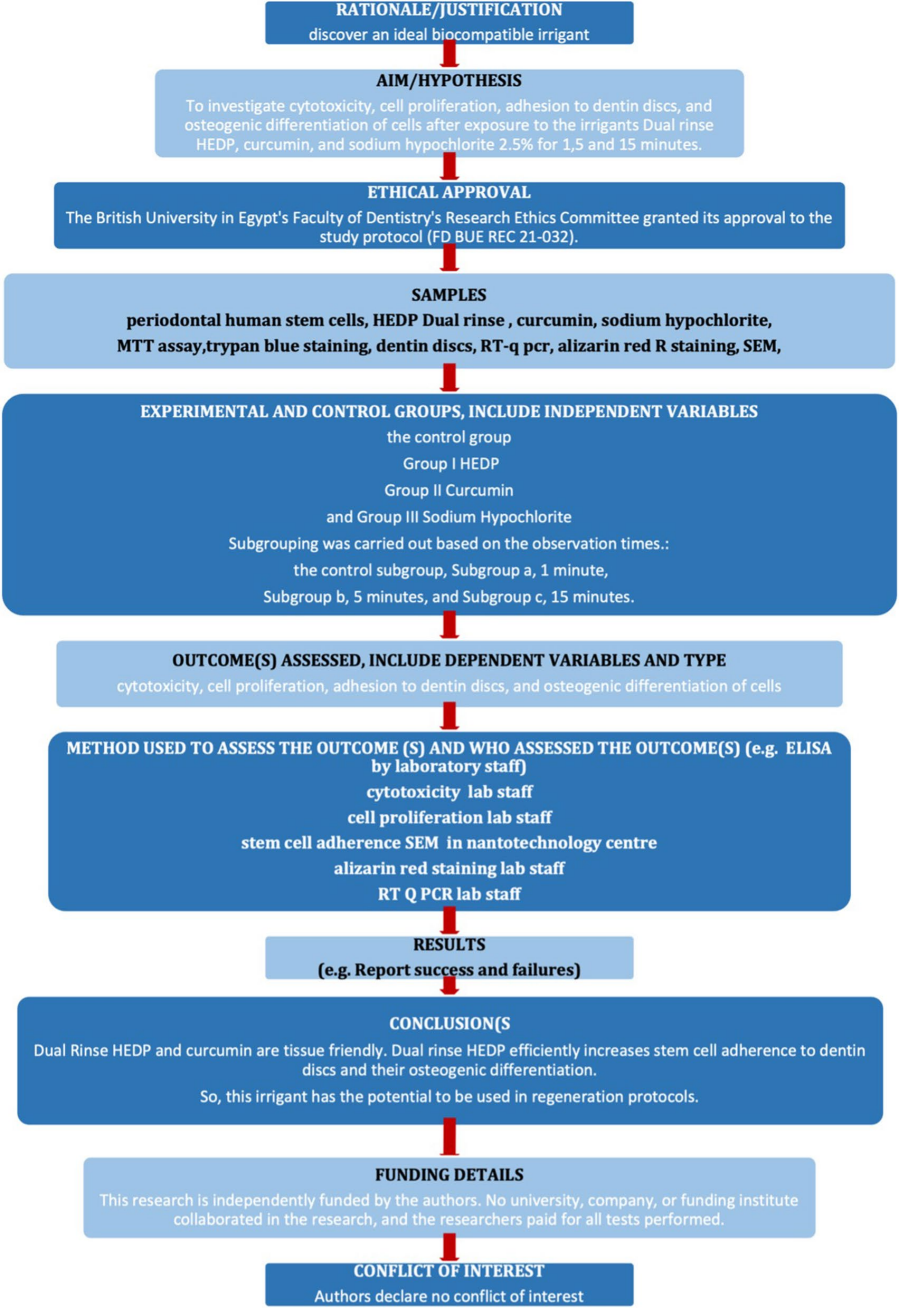
Showing laboratory flow chart of the study

All steps of the study are represented in (Fig. 1).

Consent to participate was deemed unnecessary because the teeth used in this study were extracted in the oral surgery department in the Faculty of Dentistry.

### Sample size calculation

A power analysis was conducted using an alpha (α) level of 0.05, a beta (β) level of 0.05 (corresponding to a power of 95%), and an effect size (f) of 3.02 based on findings from a prior study [27]. This analysis determined that a minimum sample size (n) of 8 samples was required. The calculation was performed with G*Power, version 3.1.9.7 [28].

### Sample grouping

Based on the material utilized, this study evaluated four types of irrigation solutions: the control group, Group I HEDP, Group II Curcumin, and Group III Sodium Hypochlorite. Subgrouping was carried out based on the observation times.: the control subgroup, Subgroup A, 1 min; Subgroup B, 5 min; and Subgroup C, 15 min.

### Samples of irrigation preparations

The manufacturer’s instructions were followed. Sodium hypochlorite was diluted to 2.5% and mixed with Dual Rinse® HEDP immediately before the beginning of the experiment [22]. Curcumin solution of concentration 2.5 mg/mL (1000 mg capsules, Puritan’s Pride, New York) [24].

## Methods

### Stem cell harvesting and culturing

Periodontal-derived mesenchymal stem cells (MSCs) were isolated in the current study from human single-rooted, single-canalled teeth extracted for orthodontic, periodontal, or prosthetic purposes. Careful scraping was done to root surfaces using a lancet to ensure the removal of all periodontal tissues attached. The extracted tissue was washed in phosphate-buffered saline PBS + penicillin–streptomycin [29]. Tissues removed from all roots were mixed, making identification of each tooth impossible, then tissues were cleaned up with phosphate-buffered saline and penicillin\streptomycin then digested with collagenase type I at 370 C. Cells were cultured in media (75 ml DMEM,15 ml FBS,5 ml Penicillin streptomycin mixture,50 µg Antifungal) and incubated at 37 0 C in 5% CO2 and 95% air by volume [29]. Cultured cells were surveyed daily for two weeks with the change of media [30]. On the twentieth day, the cultured cells showed a confluent appearance.

### Assessment

The assessment was done in two phases

#### Phase I included

- Cytotoxicity of compared irrigants using three observation periods: 1, 5, and 15 min.
- Determination of cell proliferation ‘Trypan blue’ after exposure to the compared irrigants using three observation periods: 1, 5, and 15 min.

#### Phase II included

- Stem cell adherence to treated dentin samples
- Osteogenic differentiation

Phase II proceeded after obtaining the results from Phase I, where the most acceptable exposure time to irrigants was only selected.

### Phase I

#### Assessment of sample cytotoxicity on cells (MTT protocol) [31]

Ninety-six well tissue culture plate was inoculated with 1 X 10^5^ cells/ml (100 ul/well), which equates to 10,000 cells per well, and incubated at 37 °C for 24 h to acquire a complete monolayer sheet. Cells were proven free of any physical signs of toxicity. After 24 h, the medium was replaced by tested irrigants (0.25 µl/well) for the different time intervals. At the established times, media was replaced by standard media alone. 20 µl MTT solution was placed, mixed into the media, and incubated (37 °C, 5% CO_2_) for 3 h. Formazan (MTT metabolic product) was resuspended in 20 µl/well DMSO; the formazan was blended thoroughly into the solvent. The optical density at 560 nm was measured and adjusted by subtracting the background reading at 620 nm, utilizing the Thermo Scientific™, USA, Multiskan™ Sky Microplate Spectrophotometer.

#### Determination of cell proliferation ‘Trypan blue’ [32]

A 96-well tissue culture plate was injected with cells and cultured at 37 °C for 24 h until a full monolayer sheet was formed. After 24 h, the standard medium was substituted with the tested reagents (0.25 µl/well) at various intervals. At the designated periods, media was substituted solely with standard media. Cells were collected and stained with 0.1 ml of trypan blue solution in buffer, and the total cell count and viable cell count were determined using a hemocytometer. The number of viable cells per milliliter of culture was estimated using the following formula: Viable cell count x 10^4^ × 1.1 = cells/mL culture.

### Phase II

#### Stem cell adherence to treated dentin samples [33]

##### Preparation of dentin discs

Human-impacted third molars that were non-carious were surgically extracted from healthy individuals. Teeth were preserved in sterile distilled water at 4ºC after removing organic material and calculus. The crowns were separated from the roots, and mid-coronal sections of dentine blocks were prepared to create dentin discs measuring 5 mm in diameter and 2 mm in thickness. Lastly, EDTA (5%) and NaOCl (2.5%) were used to eliminate the smear layer on dentine discs. After that, the dentin discs were split up into 4 groups. (n = 24), three of them were exposed to irrigant treatment for 5 min: Control group no treatment, Group I HEDP, Group II Curcumin, and Group III NaOCl.

Discs were sterilized with ethylene dioxide, seeded with HPSC (3 × 10^4^ cells/ml), and then suspended in their medium. As positive controls, the same number of cells were grown without dentine disks.

At various magnifications, dentin disc samples were examined using a scanning electron microscope (JEOL JSM-5200, Tokyo, Japan).

#### Osteogenic differentiation [34]

Cells were introduced into a 6-well plate at a density of 30 × 10^4^ cells per well (*n* = 3). To encourage osteogenic development, the culture media was replaced by an osteogenic medium that contained a complete culture medium enriched with 2.5 mg/l l-ascorbic acid (Sigma Aldrich, Steinheim, Germany), 10 mM beta-glycerophosphate (Merck, Darmstadt, Germany), and 0.1 μM dexamethasone. The cells were exposed to different irrigants for five minutes at the designated time. The regular culture medium and the osteogenic medium were changed every three days for two weeks. Cells cultured in an osteogenic medium comprised the positive control group, while cells grown in a culture medium were the negative control group.

#### Alizarin Red S assay

This assay was performed following the formation of mineralized nodules after 14 days of osteogenic differentiation (n = 3). The process involved washing and fixing cells with 70% ethanol for 15 min. A 40 mM solution of Alizarin Red S (Sigma-Aldrich, Steinheim, Germany) was administered and incubated for 30 min at room temperature. Extra stains were removed, and an inverted microscope was utilized to get images of the stained nodules. A 10% solution of glacial acetic acid (Sigma-Aldrich, Steinheim, Germany) was employed as a solubilizing agent to convert the red dye into a yellow tint. Using a microplate reader, the modified solution was assessed for absorbance at 405 nm. Cells cultivated in an osteogenic medium comprised the positive control group, while cells grown in an ordinary medium comprised the negative control group.

#### RT‐qPCR

The quantification of odontogenic and osteogenic gene expression was done through RT-qPCR analysis (*n* = 3) of key genes, including expression of genes Bone morphogenetic protein 2 (BMP-2), Transforming growth factor beta 1 (TGF-β1), Vascular endothelial growth factor (VEGF) and dentin sialo phosphoprotein (DSPP). The QIAGEN RNA Extraction kit (QIAGEN GmbH, Hilden, Germany) was used to extract total mRNA from each sample following the manufacturer's instructions, followed by cDNA. The resultant cDNA was amplified and quantified using SYBR Green Supermix (Bio-Rad) on an RT-qPCR machine from Bio-Rad. The levels of mRNA expression that were measured were normalized for β-actin. The primer sequences for the osteogenic and odontogenic markers are listed in Table 1. Every sample was measured three times, and the results were reported as fold change compared to the control.

**Table 1:** Primer sequences of genes used in RT-PCR

Gene	Forward sequence	Reverse sequence
***BMP-2***	TGTATCGCAGGCACTCAGGTCA	CCACTCGTTTCTGGTAGTTCTTC
***TGF-β1***	GGATACCAACTATTGCTTCAGCT	AGGCTCCAAATGTAGGGGCAGGG
***VEGF***	TGCAGATTATGCGGATCAAACC	TGCATTCACATTTGTTGTGCTGTAG
***DSPP***	GACCCCTTCATTGACCTCAACT	TGCCATTTGCTGTGATGTTT
***Β-actin***	TCCGTCGCCGGTCCACACCC	TCACCAACTGGGACGATATG

#### Statistical analysis

For cytotoxicity assessment and determination of cell proliferation, numerical data were expressed as mean ± standard deviation (SD). The Shapiro–Wilk test was employed to assess data normality, and Levene's test was used to verify the homogeneity of variances. Since the data were parametric and exhibited homogeneous variances, one-way ANOVA followed by Tukey's post hoc test was used for intergroup and subgroup comparisons. Statistical significance was set at *p* < 0.05 for all tests.

Each experiment was performed independently three times for osteogenic differentiation experiments, with all groups analyzed in triplicate. Data were presented as means ± SD. One-way ANOVA followed by Tukey’s post hoc test was used for comparisons, with statistical significance defined as *p* < 0.05. GraphPad Prism software (version 8.1.0) was used for statistical analysis.

## Results

### Phase I results

There are three observation periods and four groups. The results of phase I of the study were statistically analyzed and presented as intergroup and intragroup comparisons.

### Determination of sample cytotoxicity on cells (MTT protocol) Table 2

**Table 2:** Intergroup comparison of cell viability (%)

Time	Cell viability (%) (Mean ± SD)				*f*-value	*p*-value
	*Control*	*Group (I)*	*Group (II)*	*Group (III)*		
**1 min**	99.74 ± 7.00Ca	211.36 ± 18.23Aa	199.51 ± 8.80Aa	148.40 ± 13.06Ba	83.41	< 0.001*
**5 min**	99.71 ± 12.07Ca	223.26 ± 2.23Aa	122.80 ± 3.44Bb	86.85 ± 1.21Db	467.09	< 0.001*
**15 min**	100.90 ± 7.86Ca	135.78 ± 6.18Bb	194.65 ± 5.28Aa	67.26 ± 13.24Dc	195.33	< 0.001*
***f*** **-value**	0.03	89.92	236.10	77.44		
***p*** **-value**	**0.974**	< 0.001*	< 0.001*	< 0.001*		

Means with different upper and lowercase superscript letters within the same horizontal and vertical column respectively are significantly different

*Significant (*p* < 0.05)

#### Intergroup comparisons

Significant differences were recorded in all observation groups. At 1 and 5 min, group (I) HEDP recorded the best value (211.36 ± 18.23, 223.26 ± 2.23), followed by group (II) Curcumin (199.51 ± 8.80, 122.80 ± 3.44). But at 15 min, the best value was recorded by group (II) (194.65 ± 5.28), followed by group (I) (135.78 ± 6.18). Group III recorded the worst values at 5 and 15 min.

#### Intragroup comparisons

Regarding the control group, there was no significant difference between different subgroups. All the experimental groups showed significant differences between the subgroups. The 5-min subgroup showed the best value in group I HEDP (223.26 ± 2.23), followed by 1 min (211.36 ± 18.23). In Groups (II and III) the best value was measured after 1 min (199.51 ± 8.80,148.40 ± 13.06).

### Determination of cell proliferation ‘Trypan blue’

Table 3 and Fig. 2.

**Table 3:** Intergroup comparison of percentage of viable cells using trypan blue

Time	Cell viability (%) (Mean ± SD)				*f*-value	*p*-value
	*Control*	*Group (I)*	*Group (II)*	*Group (III)*		
**1 min**	74.24 ± 1.33C	99.57 ± 0.09Aa	98.93 ± 0.01Aa	92.33 ± 0.61Ba	1305.97	< 0.001*
**5 min**	74.24 ± 1.33D	99.89 ± 0.00Aa	89.29 ± 0.23Bb	78.71 ± 3.00Cb	242.81	< 0.001*
**15 min**	74.24 ± 1.33B	91.17 ± 1.57Ab	89.11 ± 0.15Ab	57.52 ± 6.78Cc	96.97	< 0.001*
***f*-value**	NA	149.22	6333.40	83.35		
***p*-value**	**NA**	< 0.001*	< 0.001*	< 0.001*		

Means with different upper and lowercase superscript letters within the same horizontal and vertical column respectively are significantly different

*NA* Not Applicable

*Significant (*p* < 0.05)

**Fig. 2:**
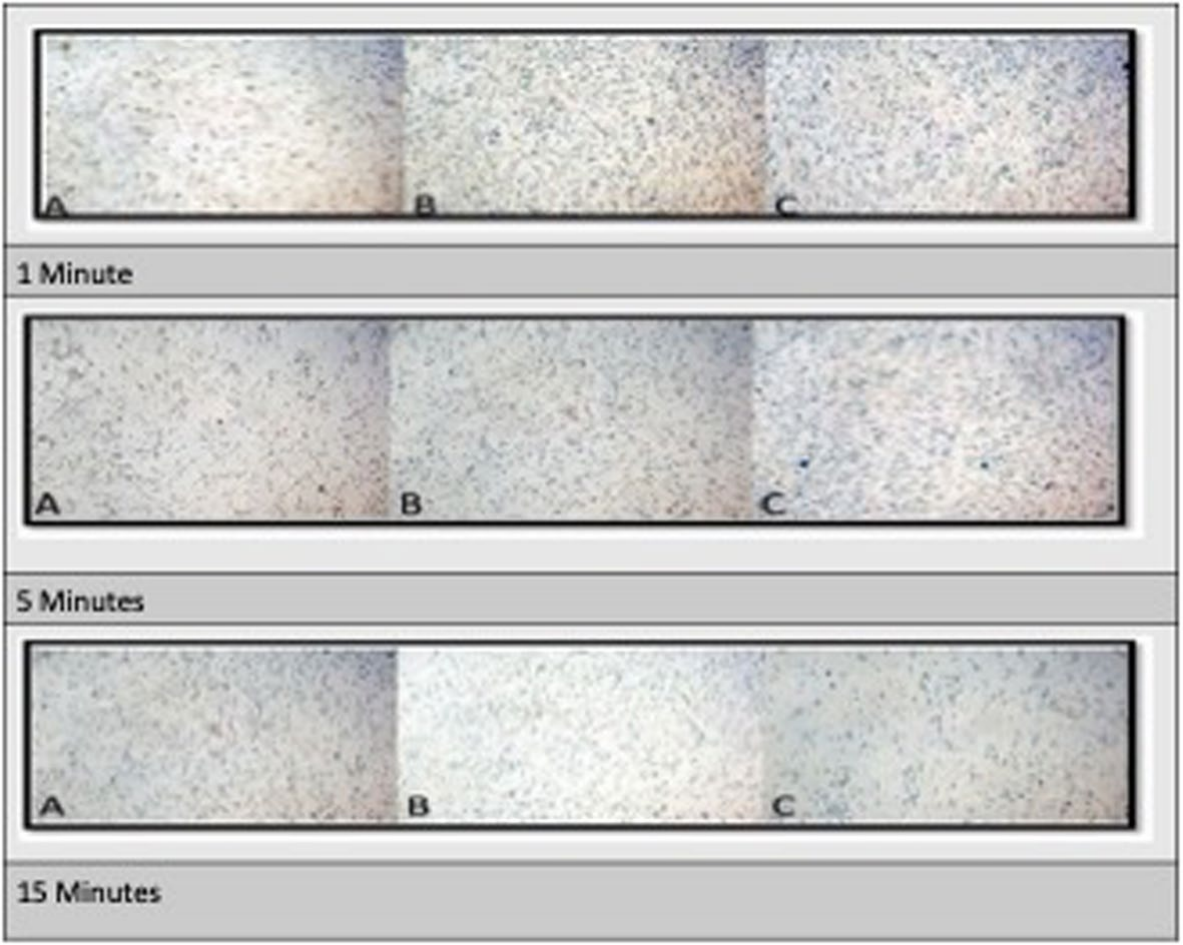
Composite figure showing photomicrographs of PDLSCs co-cultured with tested reagents for 1 min. A: PDLSCs + Group I (HEPD), B: PDLSCs + Group II ( curcumin) and C: PDLSCs + Group III NaOCl. (trypan blue × 10). 5 min. A: PDLSCs + Group I (HEPD), B: PDLSCs + Group II ( curcumin) and C: PDLSCs + Group III NaOCl. (trypan blue × 10). 15 min. A: PDLSCs + Group I (HEPD), B: PDLSCs + Group II ( curcumin) and C: PDLSCs + Group III NaOCl. (trypan blue × 10)

#### Intergroup comparisons

A significant difference was noted in all groups at all observation periods. The best value was found in group (I) HEDP, followed by group (II) Curcumin in all periods.

#### Intragroup comparisons

All subgroups showed significant differences. Group (I) HEDP recorded The best value at 5 min (99.89 ± 0.00), followed by 1 min (99.57 ± 0.09). In Groups (II and III), The best value was measured at 1 min (98.93 ± 0.01, 92.33 ± 0.61), followed by 5 min (89.29 ± 0.23, 78.71 ± 3.00).

### Phase II results

Results of phase I showed that curcumin and HEDP dual rinse performed best at 5 min of irrigation exposure, so this exposure time was selected for the assessments in phase II.

#### Stem cells adhesion to dentin (Fig. 3)

**Fig. 3:**
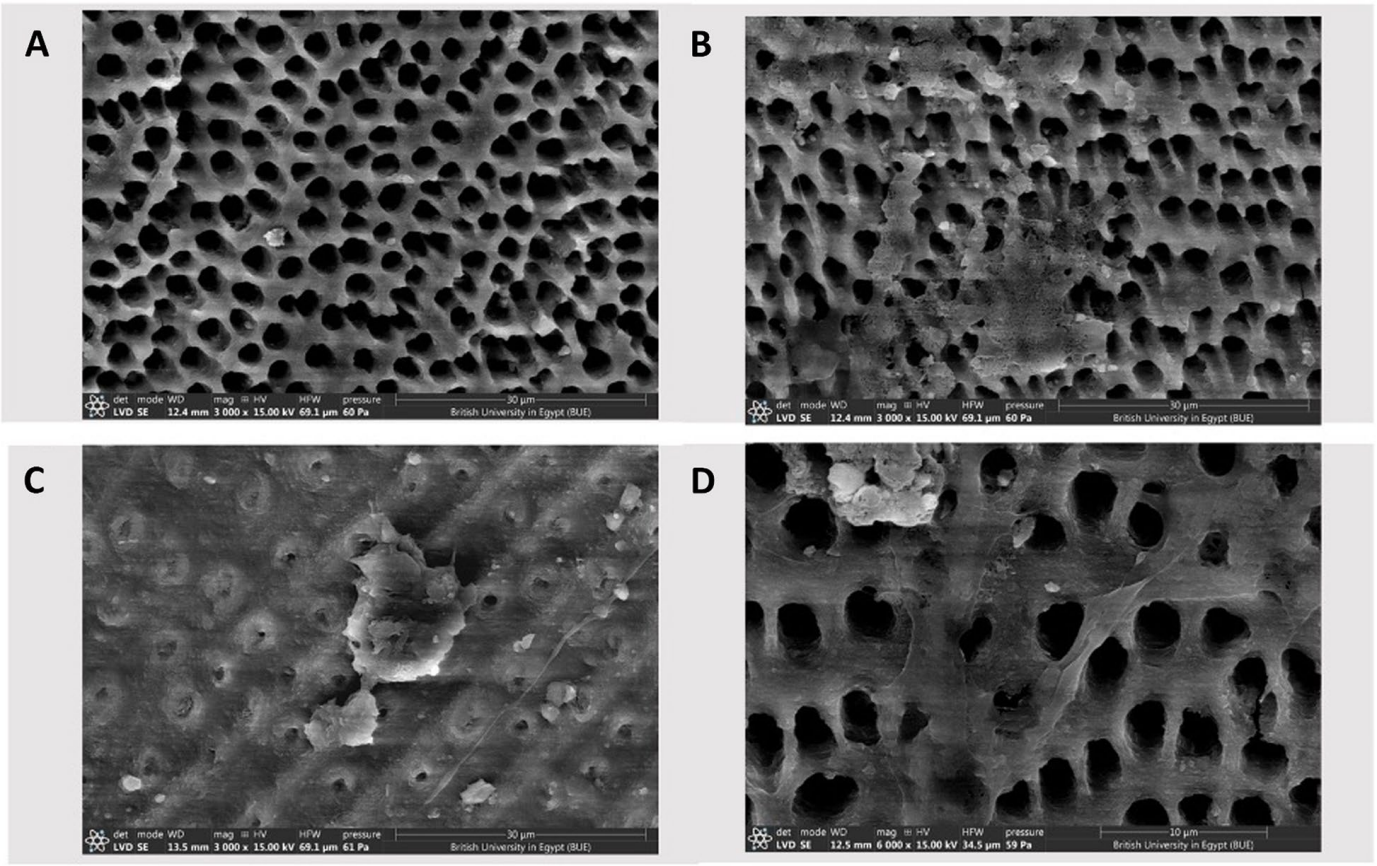
Scanning electron microscopy (SEM) of periodontal ligament stem cells (PDLSCs) cultured onto treated dentin discs. **A** The dentin surface of the control group appears with open dentinal tubules, and there is no visible attachment of cells. **B** Stem cells partially cover the dentinal tubules of the NaOCl-treated group. **C **Cluster of cells partially covering the dentin surface conditioned with curcumin. **D** Evident attachment of multiple stem cells with clear cytoplasmic extensions after HEDP treatment

The SEM examination of DPSCs attaching to dentin discs revealed notable differences among the groups. In the control group, cell attachment was minimal, with only a few cells observed on the dentin surface. Treatment with NaOCl moderately enhanced cell attachment, with more DPSCs visible and exhibiting limited spreading. Curcumin treatment further improved cell attachment, showing a higher presence of DPSCs with increased spreading and adhesion. The HEDP-conditioned group demonstrated the most pronounced improvement, with the dentin surface showing a visibly greater presence of attached DPSCs. The cells in this group exhibited extensive spreading and a distinct morphology, indicating enhanced adhesion and stronger interaction with the dentin substrate.

### Osteogenic differentiation

#### Alizarin red staining

**Fig. 4:**
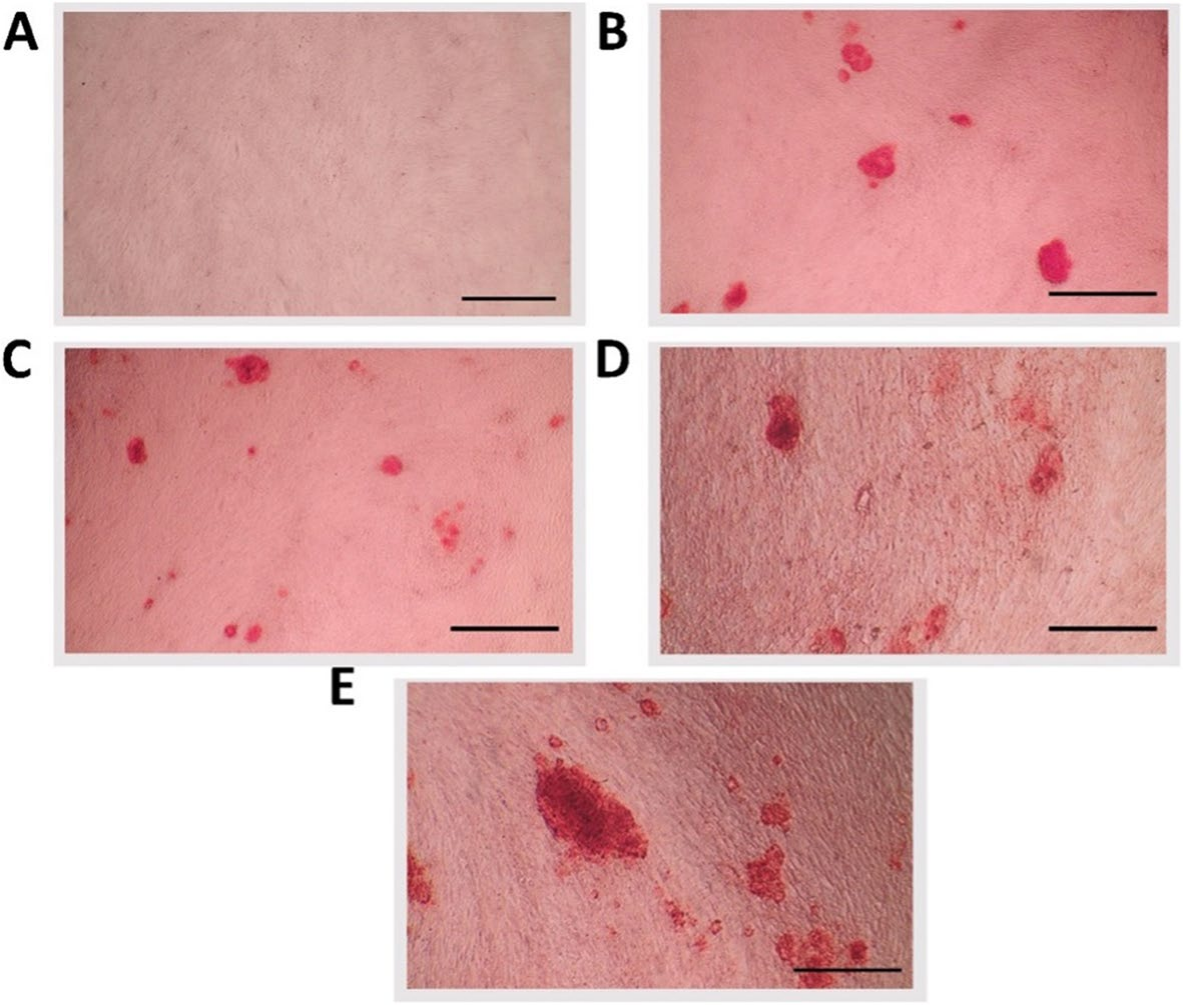
Qualitative results of Alizarin red staining of (**A**) control, (**B**) Osteogenic media, (**C**) NaOCl, (**D**) Curcumin, (**E**) HEDP

**Fig. 5:**
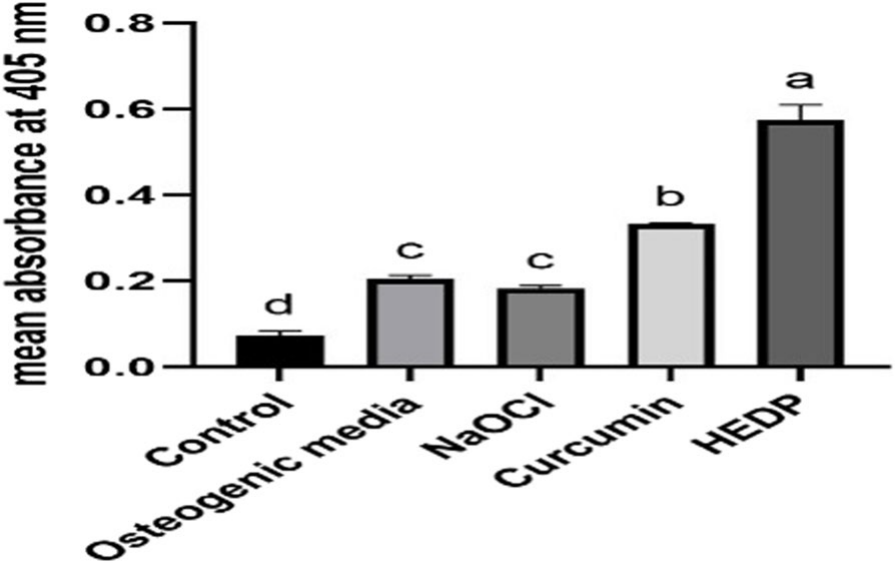
Means and standard deviation of Alizarin Red absorbance at 405 nm. Different superscript small letters indicate significant differences

Quantitative results showed that HEDP had the highest absorbance, which suggests a substantial amount of calcium deposition, indicating strong osteogenic differentiation. Curcumin had an absorbance rate higher than in control and NaOCl but lower than in the osteogenic media, suggesting that curcumin had a moderate effect on promoting calcified nodule formation (Figs. 4 and 5).

#### PCR

The findings indicate that HEDP significantly enhances the expression of genes linked to bone growth and repair, as well as angiogenesis and dentinogenesis, surpassing the effects observed in the other materials examined. The osteogenic media also induces gene upregulation, to a lesser extent, whereas NaOCl and curcumin exhibit a minor or negligible impact compared to the control group.

**Fig. 6:**
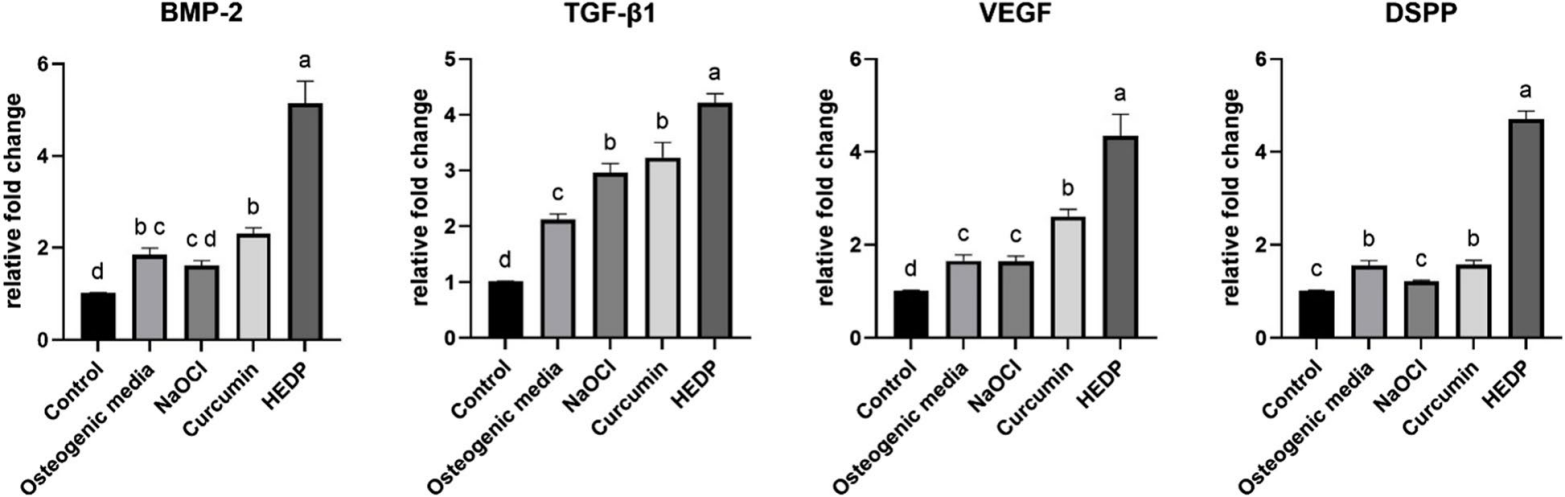
Bar graphs representing the relative fold changes in the expression of genes (BMP-2, TGF-β1, VEGF, DSPP) in cell cultures under different treatment conditions. Different superscript small letters indicate significant differences

The gene expression level of BMP-2 in the HEDP group is significantly higher than in the control, NaOCl, and curcumin groups. The same statistical significance applies to TGF-β1, VEGF, and DSPP expression levels across the different treatment conditions (Fig. 6).

## Discussion

Murray et al. 2007 proposed the term “Regenerative endodontic therapy (RET).” Revascularization is the most commonly used RET in clinical practice to encourage the maturation of immature permanent teeth. This encompasses the recruitment of undifferentiated dental stem cells and molecules from the apical region to produce a bleeding clot (BC) or using platelet-rich plasma (PRP) or platelet-rich fibrin (PRF) [35].

RET medications and disinfection irrigants impact growth factor release from dentin. Numerous biological molecules have been demonstrated to be embedded in the dentin matrix and liberated upon demineralization. These molecules include growth factors, non-collagenous proteins, and glycosaminoglycans. Before apical bleeding occurs, the dentin conditioning agent utilized in RET releases the biological cues trapped in the dentin matrix. Through apical bleeding, these biological molecules can guide the behaviors of cells organized into root canals toward pulp regeneration. Dentinogenesis is further stimulated by noncollagenous proteins such as dentin matrix protein and dentin phosphoprotein, as well as glycosaminoglycans like chondroitin sulphate and dermatan sulphate. Clinical trials have been conducted where exogenous growth factors were placed into root canals to augment the effect of endogenous dentine matrix growth factors for the regeneration of the pulp–dentine complex [36].

Avoidance of extrusion of root canal irrigation solutions into periapical tissues is inevitable during any endodontic treatment. Therefore, it is essential to perform a biochemical analysis of the effects of irrigants on the tissues they contact to anticipate and minimize any shortcomings for both the patient and the clinician. Toxicity is directly proportional to exposure time [37]. Endodontic material toxicity is a concern because irritation causes disintegration of the periradicular tissue and delays wound healing. MTT for cytotoxicity analysis of materials assesses the ability of viable cell mitochondrial dehydrogenase enzymes to change the water-soluble tetrazolium salts to the insoluble formazan crystals. MTT assesses the cytotoxicity of dental materials regarding the number of viable cells, cell metabolism, and cell morphology. Dead cells in the apoptotic phase are recognized when cellular metabolism significantly decreases [12].

The results of phase I of this study regarding the determination of sample cytotoxicity on cells (MTT protocol) in consideration of materials through the three observation periods showed that Dual rinse HEDP recorded the highest cell viability results when used for 1 min period, Curcumin gave the best results when used for 15 min and sodium hypochlorite was the most toxic for cells. The best material behavior towards cell proliferation was Group I HEDP at 5 min, Group II curcumin at 1 min, followed lastly by Group (III) at 1 min.

The results of phase I of this study regarding the determination of cell proliferation ‘Trypan blue’ come in accordance with the results of previous studies [27, 38–40], which indicated that EDTA is less cytotoxic than NaOCL and that cytotoxicity of irrigants is time dependent. It is also in accordance with the studies about curcumin cytotoxicity [41]. Cytotoxicity of curcumin to primary dental pulp fibroblasts was assessed. Curcumin promotes cell viability, potentiates primary dental pulp fibroblast growth, and can be used for vital pulp therapy.

Regarding phase II of this study, we only exposed the dentin to irrigants for 5 min, which was proven by the results in phase I of the study to be the highest results and success. The researchers focused on the successful time so as not to waste materials and cells. The scanning electron microscopy (SEM) examination of dental pulp stem cells (DPSCs) attaching to dentin discs Results contradicted other research where it was found that the difference between the cell-adhesion ability after treatment with NaOCl and curcumin was not significantly different [42].

The effect of NaOCl on cell adhesion has been a topic of extensive investigation, with the majority of studies reporting adverse outcomes due to its cytotoxic nature and disruption of extracellular matrix proteins. These effects are particularly pronounced at higher concentrations and prolonged exposure times, where NaOCl is known to significantly impair stem cell viability and adhesion.

Our findings, however, suggest that NaOCl at a lower concentration (2.5%) and controlled exposure durations may permit limited cell adhesion. This aligns with some studies reporting that reducing the concentration and minimizing exposure time can mitigate its detrimental effects. For instance, research has demonstrated that diluted NaOCl (1.5%–3%) is less harmful to stem cells, retaining some biocompatibility while maintaining antimicrobial efficacy. Such concentration-dependent variability highlights the delicate balance between achieving disinfection and preserving cellular behaviors critical for regeneration.

Osteogenic differentiation showed results in previous studies [42, 43], where all the treated groups caused increased expression of DSPP, DMP-1, OCN, and alkaline phosphatase (ALP). Also, they concluded that Curcumin had a time-dependent and dose-dependent effect on the proliferation of DPSCs and the mRNA expressions of DSPP and ALP, as well as the protein expressions of DSPP, DMP- 1, bone sialoprotein, and osteocalcin in DPSCs. ALP activity was increased in two weeks using nano curcumin on the osteogenic differentiation of DPSCs and low toxicity, making this substance fit for regenerative protocols [43–45]. According to Mumcu et al., investigations showed that HEDP caused higher growth factor release [46]. However, our results contradicted Sismanoglu et al. [47], where HEDP did not increase ALP activity significantly.

The present study's results aligned with previous research that showed that all the tested irrigants could be used in RET. Curcumin exhibited fewer adverse effects than NaOCl and EDTA due to its biocompatibility and herbal nature [42]. The toxicity of NaOCl is due to the production of the oxidizing agent hypochlorous acid (HOCL), a solvent releasing chlorine from organic tissue. Chlorine triggers the production of free radicals, increasing reactive oxygen species (ROS). A high Ph of sodium hypochlorite causes the release of hydroxyl ions. These ions cause damage in the form of channels in the cytoplasmic membrane of mitochondria, known as the mitochondrial permeability transition pore. Oxidative phosphorylation and reduced Adenosine triphosphate (ATP) occur due to the presence of the channels. Increased ROS will cause cell oxidative stress, lipid peroxidation, and diminished protein synthesis and DNA damage. These effects and reduced ATP cause cell death [48].

Like EDTA and citric acid, HEDP is a chelator but forms weaker complexes with calcium. HEDP is added to the NaOCl irrigating solution during the entire endodontic procedure. The calcium ions are continuously engaged in a complex form, so the alternating irrigation concept can be discarded [18]. It was suggested that EDTA be used along with NaOCl interchangeably for smear layer removal. EDTA reduced the antibacterial effect of NaOCl and its ability to dissolve tissues. Using EDTA repeatedly causes erosion of root canal dentine, decalcification of peritubular dentin, and exposed organic matrix dissolution. Dual Rinse HEDP was presented as a moderate alternative to EDTA as it slightly interferes with the properties of dentin [49].

The adopted hypothesis was accepted for this research and proved that the tested irrigants HEDP and curcumin had a biocompatible effect in all aspects tested and can be a potential part of the revascularization protocol.

## Conclusion

Dual Rinse HEDP and curcumin are tissue-friendly materials. Dual rinse HEDP efficiently increases stem cell adherence to dentin discs and their osteogenic differentiation.

So, this irrigant has the potential to be used in regeneration protocols.

### Limitations of the study

While valuable, this ex-vivo study's findings may not fully reflect in vivo conditions due to factors like immune response and tissue interactions. Caution is needed when extrapolating to clinical scenarios. Future research should validate these results in animal models, assess long-term regenerative effects, and explore synergistic use with growth factors and scaffolds. Comparative studies with standard agents like EDTA are also recommended to enhance clinical relevance.

## Data Availability

The datasets used and/or analysed during the current study are available from the corresponding author on reasonable request.

## Abbreviations

ALP: Alkaline phosphatase
BC: Bleeding clot
BMP-2: Bone morphogenetic protein 2
DPSCs: Dental pulp stem cells
DSPP: Dentin sialo phosphoprotein
HEDP: (1-Hydroxyethylidene-1,1-diphosphonic acid)
HPDLFc: Human periodontal ligament fibroblasts
MSCs: Mesenchymal stem cells
MTT: 3- (4.5-dimethylthiaziol-2yl) -2.5- diphenyl-tetrazolium bromide
NaOCl: Sodium hypochlorite
PRF: Platelet-rich fibrin
PRP: Platelet-rich plasma
REP: Regenerative endodontics protocol
RET: Regenerative endodontic therapy
ROS: Reactive oxygen species
SD: Standard deviation
SEM: Scanning electron microscopy
STDF: Science, Technology & Innovation Funding Authority
TGF-β1: Transforming growth factor beta 1
VEGF: Vascular endothelial growth factor
